# Localised estimates and spatial mapping of poverty incidence in the state of Bihar in India—An application of small area estimation techniques

**DOI:** 10.1371/journal.pone.0198502

**Published:** 2018-06-07

**Authors:** Hukum Chandra, Kaustav Aditya, U. C. Sud

**Affiliations:** ICAR-Indian Agricultural Statistics Research Institute, New Delhi, India; University of Pisa, ITALY

## Abstract

Poverty affects many people, but the ramifications and impacts affect all aspects of society. Information about the incidence of poverty is therefore an important parameter of the population for policy analysis and decision making. In order to provide specific, targeted solutions when addressing poverty disadvantage small area statistics are needed. Surveys are typically designed and planned to produce reliable estimates of population characteristics of interest mainly at higher geographic area such as national and state level. Sample sizes are usually not large enough to provide reliable estimates for disaggregated analysis. In many instances estimates are required for areas of the population for which the survey providing the data was unplanned. Then, for areas with small sample sizes, direct survey estimation of population characteristics based only on the data available from the particular area tends to be unreliable. This paper describes an application of small area estimation (SAE) approach to improve the precision of estimates of poverty incidence at district level in the State of Bihar in India by linking data from the Household Consumer Expenditure Survey 2011–12 of NSSO and the Population Census 2011. The results show that the district level estimates generated by SAE method are more precise and representative. In contrast, the direct survey estimates based on survey data alone are less stable.

## Introduction

Bihar is third-most populous state in India. According to the 2011 Population Census, the population of state is 103 million, which is about 8.58 percent of the total population of the country. Poverty is a very complex issue in Bihar and there is an exigent need to devise a focused strategy for poverty eradication. Reliable, qualitative and timely disaggregate level data is essential for effective planning, implementation and monitoring of various Government schemes in Bihar. Spatially disaggregated level data is inevitable for identifying the areas more in need and for developing focused and target oriented intervention programs. The geographic distribution of poverty and wealth is used to make decisions about resource allocation and provides a foundation for the study of inequality and the determinants of economic growth [1–2]. In developing countries, however, the scarcity of reliable quantitative data represents a major challenge to policy-makers and researchers. In India, National Sample Survey Office (NSSO) surveys are the main source of official statistics. A range of invaluable data at state and national level are generated through these surveys. The state level estimates generated by these surveys often masked the local level heterogeneity. More importantly, state level estimates do not adequately capture the extent of geographical inequalities which restricts the scope for evaluating progress locally within and between administrative units. But, the NSSO survey data cannot directly be used to produce reliable disaggregate level (e.g. district or further disaggregate level) estimates due to small sample sizes. In the survey literature, an area (or domain) is regarded as small if the area-specific (or domain-specific) sample is not large enough to support a direct survey estimator of adequate precision with unacceptably large coefficient of variation [3–4]. At the same time it is also true that conducting district specific survey is going to be very trivial and costly as well as time consuming job. An alternative solution to this problem is to use small area estimation (SAE) techniques. The SAE approach produces reliable estimates for such small areas with small sample sizes by borrowing strength from data of other areas. The SAE techniques are based on model-based survey estimation methods. The idea is to use statistical models to link the variable of interest with auxiliary information, e.g. Census and Administrative data, for the small areas to define model-based estimators for these areas. In other words, the SAE method uses indirect small area estimators that make use of the sample data from related areas or domains through linking models, and hence increases the effective sample size in the small areas. Such estimators can provide significantly smaller coefficient of variation than direct estimators, provided the linking models are valid, see [5]. Recently, some researchers have also used satellite imagery and mobile phone networks data to predict the poverty. Existing high resolution daytime satellite imagery is used to predict the spatial distribution of economic well-being across five African countries namely Nigeria, Tanzania, Uganda, Malawi, and Rwanda [6]. Anonymized data from mobile phone networks, combined with survey data, are also used to predict the poverty and wealth of individual subscribers, as well as to create high-resolution maps of the geographic distribution of wealth [7].

Based on the level of auxiliary information available from secondary data sources, SAE methods are categorized as based on area level and unit level small area models. Area level small area models are used when auxiliary information is available only at area (or aggregated) level. They relate area-specific direct survey estimates to area-specific covariates [8]. Unit level small area models, proposed originally by [9], relate the unit values of a study variable to unit-specific covariates. In this paper, we consider the area level version of small area model since auxiliary variables (covariates) are available only at the area (or aggregated) level. We apply SAE techniques to produce reliable small area estimates of the poverty incidence at district level in the State of Bihar in India by linking data from the existing Household Consumer Expenditure Survey data and the Population Census. Small areas are defined as the different districts of State of Bihar in India. In addition, poverty map is also produce to show spatial inequality in distribution of poverty incidence in the state. This paper, in particular, illustrates and provides a guidelines on how the existing large scale survey and Census data can be linked to generate reliable small area estimates for various policy relevant parameters.

## Data sources and model specification

This Section describes basic sources of data i.e. survey data and the auxiliary data used to estimate the poverty incidence at district level. The poverty incidence is defined as the proportion of households with income below the poverty line, also referred as head count ratio (HCR). The HCR is a poverty indicator which measures the frequency of households under poverty line. Two types of variables are required for SAE analysis, the variable of interest and the auxiliary variables. In this study, the variable of interest for which small area estimates are required is drawn from the Household Consumer Expenditure Survey 2011–12 of NSSO for rural areas of the State of Bihar in India. The NSSO survey data is not freely downloadable but it can be obtained from the NSSO, Ministry of Statistics and Programme Implementation, Government of India (http://mospi.nic.in/). The sampling design used in the NSSO data is stratified multi-stage random sampling with districts as strata, villages as first stage units and households as the second stage units. A total of 3312 households were surveyed from the 38 districts of the Bihar. The district-wise sample size varied from minimum 64 to maximum 128 with average of 87 (Table 1). From Table 1, it is evident that district level sample sizes are very small with very low values of average sampling fraction of 0.00025. Therefore, it is difficult to produce reliable estimates of the poverty incidence and their standard errors at district level. Hence, the application of SAE technique is an obvious choice for obtaining the district level estimates of poverty incidence. The SAE technique is expected to provide reliable estimates for the districts having small sample data [3–5]. The target variable used for the study is poor households. The poverty line has been used to identify whether given household is poor or not. A household having monthly per capita consumer expenditure below the state’s poverty line (Rs 778) is categorised as poor household. The poverty line used in this study is same as those of year 2011–12, given by the planning commission, Government of India (see http://planningcommission.nic.in/news/press_pov2307.pdf).

**Table 1:** Distribution of district wise sample sizes (*n*), estimates of poverty incidence (estimate) along 95% confidence interval (95% CI) and percentage coefficient of variation (% CV) generated by direct survey estimate (DIR) and model based small area estimate (SAE estimate) for Bihar.

District	*n*	DIR estimate				SAE estimate			
		Estimate	95% CI		% CV	Estimate	95% CI		% CV
			Lower	Upper			Lower	Upper	
**Pashchim Champaran**	96	0.34	0.25	0.44	14.55	0.33	0.24	0.42	14.06
**Purba Champaran**	128	0.13	0.07	0.18	24.00	0.14	0.08	0.19	20.50
**Sheohar**	64	0.30	0.18	0.41	20.21	0.28	0.18	0.38	18.34
**Sitamarhi**	96	0.38	0.28	0.47	13.33	0.36	0.27	0.45	12.96
**Madhubani**	128	0.10	0.05	0.15	29.54	0.12	0.06	0.17	22.81
**Supaul**	64	0.05	-0.01	0.10	64.00	0.09	0.03	0.14	33.28
**Araria**	96	0.07	0.02	0.13	41.14	0.10	0.04	0.15	27.56
**Kishanganj**	64	0.09	0.02	0.17	42.67	0.12	0.05	0.19	29.11
**Purnia**	88	0.27	0.18	0.37	18.33	0.26	0.18	0.35	16.94
**Katihar**	88	0.18	0.10	0.26	22.00	0.19	0.11	0.26	20.94
**Madhepura**	64	0.00	-	-	-	0.06	0.02	0.10	37.27
**Saharsa**	64	0.08	0.01	0.14	38.40	0.11	0.04	0.17	31.00
**Darbhanga**	128	0.23	0.16	0.31	17.07	0.23	0.16	0.30	15.47
**Muzaffarpur**	128	0.23	0.16	0.31	17.07	0.23	0.16	0.29	15.33
**Gopalganj**	96	0.21	0.13	0.29	19.20	0.20	0.13	0.28	19.17
**Siwan**	96	0.29	0.20	0.38	17.14	0.28	0.20	0.37	15.40
**Saran**	128	0.16	0.09	0.22	19.20	0.16	0.10	0.22	18.75
**Vaishali**	96	0.09	0.04	0.15	32.00	0.12	0.06	0.18	25.64
**Samastipur**	128	0.17	0.11	0.24	17.45	0.18	0.11	0.24	17.97
**Begusarai**	96	0.06	0.01	0.11	32.00	0.09	0.04	0.14	29.73
**Khagaria**	64	0.09	0.02	0.17	42.67	0.12	0.05	0.19	28.87
**Bhagalpur**	96	0.18	0.10	0.25	22.59	0.18	0.11	0.25	20.14
**Banka**	64	0.22	0.12	0.32	22.86	0.22	0.13	0.31	21.42
**Munger**	64	0.27	0.16	0.38	22.59	0.25	0.15	0.35	20.08
**Lakhisarai**	64	0.16	0.07	0.25	32.00	0.16	0.08	0.23	25.64
**Sheikhpura**	64	0.17	0.08	0.27	29.09	0.17	0.09	0.25	24.96
**Nalanda**	96	0.29	0.20	0.38	17.14	0.28	0.19	0.36	15.85
**Patna**	96	0.30	0.21	0.39	16.55	0.29	0.21	0.38	14.98
**Bhojpur**	96	0.38	0.28	0.47	13.33	0.35	0.26	0.44	13.06
**Buxar**	64	0.34	0.23	0.46	17.45	0.31	0.20	0.41	17.43
**Kaimur**	64	0.23	0.13	0.34	21.33	0.22	0.13	0.31	21.03
**Rohtas**	96	0.33	0.24	0.43	15.00	0.31	0.22	0.40	14.47
**Jehanabad**	64	0.27	0.16	0.38	22.59	0.26	0.16	0.35	19.53
**Aurangabad**	64	0.19	0.09	0.28	26.67	0.19	0.11	0.28	23.17
**Gaya**	128	0.20	0.13	0.26	20.48	0.19	0.13	0.26	17.27
**Nawada**	64	0.16	0.07	0.25	32.00	0.16	0.08	0.24	25.45
**Jamui**	64	0.39	0.27	0.51	15.36	0.34	0.24	0.45	15.92
**Arwal**	64	0.20	0.10	0.30	24.62	0.19	0.10	0.28	23.79

The auxiliary (covariates) variables used in this analysis are drawn from the Population Census 2011. The Population Census 2011 data can be accessed freely from the Census of India website: http://www.censusindia.gov.in/2011census/population_enumeration.html. These auxiliary variables are only available as counts at district level, and there are approximately 50 such covariates that are available for use in SAE analysis. We therefore carried out a preliminary data analysis in order to define appropriate covariates for SAE modelling, using Principal Component Analysis (PCA) to derive composite scores for selected groups of variables. The reader is referred to [10–11] for a more detailed discussion on PCA. The PCA variables (i.e. composite scores derived for selected groups of variables using PCA) instead of Census variables are often used in model-based small area estimation method because maximum variability is explained with reduced dimension of auxiliary variables, see for example [12–13]. We carried out PCA separately on three groups of variables, all measured at district level and identified as *X*_1_, *X*_2_ and *X*_3_ respectively. The PCA was done in SPSS software. The first group (*X*_1_) consisted of literacy rates by gender and proportions of worker population by gender. The first principal component for this group (*X*_11_) explained 52% of the variability in the *X*_1_ group, while adding the second principal component (*X*_12_) increased this to 100%. The second group (*X*_2_) consisted of the proportions of main worker by gender, proportions of main cultivator by gender and proportions of main agricultural labourer by gender. The first principal component (*X*_21_) for this second group explained 67% of the variability in the *X*_2_ group, while adding the second component (*X*_22_) increased this to 94%. Finally, the third group (*X*_3_) consisted of proportions of marginal cultivator by gender and proportions of marginal agriculture labourers by gender. The first principal component (*X*_31_) for this third group explained 52% of the variability in the *X*_3_ group, while adding the second component (*X*_32_) increased this to 77%. We then fitted a generalised linear model using direct survey estimates of proportions of poor households as the response variable and the six principal component scores *X*_11_,*X*_12_,*X*_21_,*X*_22_,*X*_31_, and *X*_32_ as potential covariates. The final selected model included the three covariates *X*_11_, *X*_21_ and *X*_31_. This final model was then used to produce district wise estimates of poverty incidence, i.e. estimates of the head count ratio (HCR) used in poverty mapping. This model was fitted using the glm() function in R and specifying the family as “binomial” and the district specific sample sizes as the weight.

## Methodology

In this Section we illustrate the theoretical framework used to produce small area estimates of the poverty incidence and their measure of precision. The details presented here are followed from [12–13]. Let us assume a finite population *U* of size *N* and a sample *s* of size *n* is drawn from this population with a given survey design. We assume that this population consists of *D* small areas or small domains (or simply areas or domains) *U*_*d*_(*d* = 1,…,*D*) such that $U=\cup_{d=1}^{D}U_{d}$ and $N=\sum_{d=1}^{D}N_{d}$. Throughout, we use a subscript *d* to index the quantities belonging to small area *d* (*d* = 1,…,*D*), where *D* is the number of small areas (or areas) in the population. The subscript *s* and *r* are used for denoting the quantities related to the sample and non-sample parts of the population. So that *n*_*d*_ and *N*_*d*_ represent the sample and population (i.e., number of households in sample and population) sizes in district *d*, respectively. Let *s*_*d*_ denotes the part of sample from area *d* such that $s=\cup_{d=1}^{D}s_{d}$ and $n=\sum_{d=1}^{D}n_{d}.$ Let *y*_*di*_ denotes the value of target variable of interest *y* for unit *i* in small area *d*. Let assume that the variable of interest *y* is binary and the target is the estimation of population counts $y_{d}=\sum_{i\in U_{d}}y_{di}$ or population proportions $P_{d}=N_{d}^{-1}\left(\sum_{i\in U_{d}}y_{di}\right)$ in area *d*. The direct estimator of proportion of poor household is defined as ${\hat{p}}_{d}^{Direct}={\left(\sum_{i\in s_{d}}^{}w_{di}\right)}^{-1}\left(\sum_{i\in s_{d}}^{}w_{di}y_{di}\right)$, where *w*_*di*_ is the survey weight associated with household *i* in area *d*. Assuming that joint inclusion 1/*w*_*di*,*d*′*j*_ = 0 for *d* ≠ *d*′ or *i* ≠ *j*, the estimate of variance of ${\hat{p}}_{d}^{Direct}$ is $v({\hat{p}}_{d}^{Direct})={\left(\sum_{i\in s_{d}}^{}w_{di}\right)}^{-2}\left\{\sum_{i\in s_{d}}^{}w_{di}(w_{di}-1){\left(y_{di}-{\hat{p}}_{d}^{Direct}\right)}^{2}\right\}$, see for example [14]. Let us denote by *y*_*sd*_ and *y*_*rd*_ the sample and non-sample counts of poor households in area (or district) *d*. The sample count *y*_*sd*_ has a Binomial distribution with parameters *n*_*d*_ and *p*_*d*_, denoted by *y*_*sd*_ ~ *Bin*(*n*_*d*_,*p*_*d*_), where *p*_*d*_ is the probability of a poor household in area *d*, often termed as the probability of a ‘success’. Similarly, *y*_*rd*_ ~ *Bin*(*N*_*d*_ − *n*_*d*_,*p*_*d*_). Further, *y*_*sd*_ and *y*_*rd*_ are assumed to be independent Binomial variables with *p*_*d*_ being a common success probability. Here we assume that only aggregated level data is available for the small area modelling. For example, from survey data *y*_*sd*_ and from secondary data sources (i.e. Census and administrative records etc) **x**_*d*_, the *p*-vector of the covariates, are available for area *d*. Following [12–13], the model linking the probabilities of success *p*_*d*_ with the covariates **x**_*d*_ is the logistic linear mixed model given by

$$logit(p_{d})=\ln\left\{\frac{p_{d}}{1-p_{d}}\right\}=\eta_{d}=x_{d}^{T}\beta +u_{d},$$ (1)

with $p_{d}=\exp(x_{d}^{T}\beta +u_{d}){\left\{1+\exp(x_{d}^{T}\beta +u_{d})\right\}}^{-1}=\mathrm{expit}(x_{d}^{T}\beta +u_{d})$. Here **β** is the *p*-vector of regression coefficients, often known as fixed effect parameters, and *u*_*d*_ is the area-specific random effect that capture the between area heterogeneity. We assume that *u*_*d*_’s are independent and normally distributed with mean zero and variance *ϕ*. Here, we observe that equation number (1) relates the area (or district) level proportions (direct estimates) from the survey data to the area (or district) level covariates. This type of model is often referred to as ‘area-level’ model in SAE terminology, see for example [4, 8]. Area level model was originally proposed by Fay and Herriot [8] for the prediction of mean per-capita income (PCI) in small geographical areas (less than 500 persons) within counties in the United States. Fay-Herriot model [8] is widely used area level model for the estimation of small area quantities. In many small area applications, when data are non-linear on original scale, Fay-Herriot model is fitted on transformed scale. For example, some function of small area direct survey estimates is linearly related to the area aggregates of auxiliary variables. In small area income and poverty estimation project of the US Census Bureau, namely SAIPE, Fay-Herriot model is fitted using logarithm of direct poverty rate estimates [15]. Similarly, in Chilean poverty estimation methodology, Fay-Herriot model is fitted with transformed poverty rate estimates using the arcsine transformation [16]. In such cases, model parameters are estimated under Fay-Herriot model fitted on transformed scale. This is followed by back transformation to obtain the estimate for small area quantities on original scale. However, back transformation leads to biased estimates of small area quantities on original scale [15, 17]. This approach of poverty estimation based on Fay-Herriot method using the transformed direct estimates is often criticised. The Fay-Herriot method for SAE is based on area level linear mixed model and their approach is applicable to a continuous variable. This model is not applicable for non-normal data. Equation number (1) on the other hand, a special case of a generalized linear mixed model (GLMM) with logit link function, is suitable for modelling discrete data, particularly the binary variables. Here,

$$y_{ds}|u_{d}∼\mathrm{Binomial}\;\left(n_{d},\mathrm{expit}(x_{d}^{T}\beta +u_{d})\right)\;\mathrm{and}\;y_{dr}|u_{d}∼\mathrm{Binomial}\;\left(N_{d}-n_{d},\mathrm{expit}(x_{d}^{T}\beta +u_{d})\right).$$

This leads to $E\left(y_{sd}|u_{d}\right)=n_{d}\mathrm{expit}(x_{d}^{T}\beta +u_{d})$ and $E\left(y_{rd}|u_{d}\right)=\left(N_{d}-n_{d}\right)\;\mathrm{expit}(x_{d}^{T}\beta +u_{d})$. Collecting the area level models given by equation number (1), we can write population level version of model of form

g(p)=η=Xβ+Zu. (2)

Here p = (*p*_1_,…,*p*_*D*_)^*T*^, $X=(x_{1}^{T},\ldots .,x_{D}^{T}{)}^{T}$ is a *D*×*p* matrix, **Z** is a *D*×*D* diagonal matrix and **u** = (*u*_1_,…,*u*_*D*_)^*T*^ is a vector of *D*×1 of area random effects, which is normally distributed with mean zero and variance **Ω** = *ϕ***I**_*D*_. Here, **I**_*D*_ is a *D*×*D* diagonal matrix. Note that estimation of fixed effect parameters **β** and area specific random effects *u*_*d*_’s uses the data from all small areas. We used an iterative procedure that combines the Penalized Quasi-Likelihood (PQL) estimation of **β** and **u** with restricted maximum likelihood (REML) estimation of *ϕ* to estimate these unknown parameters. Detailed description of the approach can be followed from [18–20]. Let us write the total counts, i.e. the total number of poor households in district *d* as *y*_*d*_ = *y*_*sd*_ + *y*_*rd*_, where *y*_*sd*_ (sample count) is known and *y*_*rd*_ (non-sample count) is unknown. Therefore, a plug-in empirical best predictor (EBP) estimate of the total count in area (or district) *d*, obtained by replacing *y*_*rd*_ by its predicted value, is given by

$${\hat{y}}_{d}^{EBP}=y_{sd}+\hat{E}\left(y_{rd}|u_{d}\right)=y_{sd}+\left(N_{d}-n_{d}\right)\left[\mathrm{expit}(x_{d}^{T}\hat{\beta }+Z_{d}^{T}\hat{u})\right],$$ (3)

where $Z_{d}^{T}=\left(0,..,1,.,0\right)$ is 1×*D* vector with 1 in position *d*-th. An estimate of proportion in area *d* is then obtained as ${\hat{p}}_{d}^{EBP}=N_{d}^{-1}{\hat{y}}_{d}^{EBP}$. For area with zero sample sizes (i.e. non-sampled areas), the conventional approach for estimating area proportions or counts is synthetic estimation, based on a suitable GLMM fitted to the data from the sampled areas [12]. From equation number (1), for non-sampled areas, the synthetic type predictor of total count for area *d* is ${\hat{y}}_{d}^{SYN}=N_{d}\mathrm{expit}(x_{d,out}^{T}\hat{\beta })$, where **x**_*d*,*out*_ denote the vector of covariates associated with non-sampled area *d*. An alternative to predictor (3) has been proposed by [21]. Unfortunately, this predictor does not have a closed form and can only be computed via numerical approximation. This is generally not straightforward, and so many users tend to favour computation of a plug-in empirical predictors like (3). There are several alternative approaches for estimating the small area counts. For example, Bayesian approaches for modelling the counts, using a negative binomial distribution or via a hierarchical Poisson-gamma model, are popular in the disease mapping and ecological regression literature, see for example, [22–26] and references therein.

The mean squared error (MSE) estimates are computed to assess the reliability of estimates and also to construct the confidence interval for the estimates. Following [12, 13, 19, 20], the MSE estimate of small area predictor (3) is given by

$$mse({\hat{p}}_{d}^{EBP})=m_{1}(\hat{\phi })+m_{2}(\hat{\phi })+2m_{3}(\hat{\phi }).$$ (4)

In equation number (4), the first two components *m*_1_ and *m*_2_ constitute the largest part of the overall MSE estimate. These are the MSE of the best linear unbiased predictor type estimator when variance component parameter *ϕ* is known [20]. The third component *m*_*3*_ is the variability due to the estimate of *ϕ*. For simplicity and ease of implementation, we define few notations to express different components of the MSE estimate given in equation number (4). We denote by ${\hat{V}}_{sd}=diag\;\left\{n_{d}{\hat{p}}_{d}^{EBP}(1-{\hat{p}}_{d}^{EBP})\right\}$ and ${\hat{V}}_{rd}=diag\;\left\{\left(N_{d}-n_{d}){\hat{p}}_{d}^{EBP}(1-{\hat{p}}_{d}^{EBP}\right)\right\}$, the diagonal matrices defined by the corresponding variances of the sample and non-sample part respectively. We then define $A=\left\{diag(N_{d}^{-1})\right\}{\hat{V}}_{rd}$, $B=\left\{diag(N_{d}^{-1})\right\}\left\{{\hat{V}}_{rd}X-A\hat{\Sigma }{\hat{V}}_{sd}X\right\}$ and $\hat{\Sigma }={\left(\phi^{-1}I_{D}+{\hat{V}}_{sd}\right)}^{-1}$, where **I**_*D*_ is an identity matrix of order *D*. We further write ${\hat{V}}_{\left(1\right)}={\left\{X^{T}{\hat{V}}_{sd}X-X^{T}{\hat{V}}_{sd}\hat{\Sigma }{\hat{V}}_{sd}X\right\}}^{-1}$ and ${\hat{V}}_{\left(2\right)}=\hat{\Sigma }+\hat{\Sigma }{\hat{V}}_{sd}X{\hat{V}}_{\left(1\right)}X^{T}{\hat{V}}_{sd}^{T}\hat{\Sigma }$. Using these notations, the various components of MSE estimate are:

$$m_{1}(\hat{\phi })=A\hat{\Sigma }A^{T},\;m_{2}(\hat{\phi })=B{\hat{V}}_{\left(1\right)}B^{T},\;\mathrm{and}\;m_{3}(\hat{\phi })=trace\left({\hat{\nabla }}_{i}{\hat{\Sigma }}^{+}{\hat{\nabla }}_{j}^{T}v(\hat{\phi })\right)\;\mathrm{with}\;{\hat{\Sigma }}^{+}={\hat{V}}_{sd}+\hat{\phi }I_{D}{\hat{V}}_{sd}{\hat{V}}_{sd}^{T}.$$

Here $v(\hat{\phi })$ is the asymptotic covariance matrix of the estimate of variance component $\hat{\phi }$, which can be evaluated as the inverse of the appropriate Fisher information matrix for $\hat{\phi }$. Further, this also depends upon whether we are use ML or REML estimate for $\hat{\phi }$. We use REML estimate for $\hat{\phi }$, then $v(\hat{\phi })=2{\left({\hat{\phi }}^{-2}(D-2a_{1})+{\hat{\phi }}^{-4}a_{11}\right)}^{-1}$ with $a_{1}={\hat{\phi }}^{-1}trace({\hat{V}}_{\left(2\right)})$ and $a_{11}=trace({\hat{V}}_{\left(2\right)}{\hat{V}}_{\left(2\right)})$. Finally, $\Delta =A\hat{\Sigma }$ and ${\hat{\nabla }}_{i}={\partial (\Delta_{i})/\partial \phi |}_{\phi =\hat{\phi }}={\partial (A_{i}\hat{\Sigma })/\partial \phi |}_{\phi =\hat{\phi }}$, where **A**_*i*_ is the *i*^*th*^ row of the matrix **A**. The empirical results reported in the next Sections are obtained using R software.

## Results and discussion

We now discuss the results (i.e. estimates of the proportion of poor households at district level in the State of Bihar) generated by the model-based small area method (3). In this analysis, we use survey data from the Household Consumer Expenditure Survey 2011–12 of NSSO and the Population Census 2011, and assume a binomial specification for the observed district level sample counts. Model specification for this application was discussed in previous Section, and resulted in the identification of three PCA-based covariates, labelled *X*_11_,*X*_21_ and *X*_31_, there.

**Fig 1:**
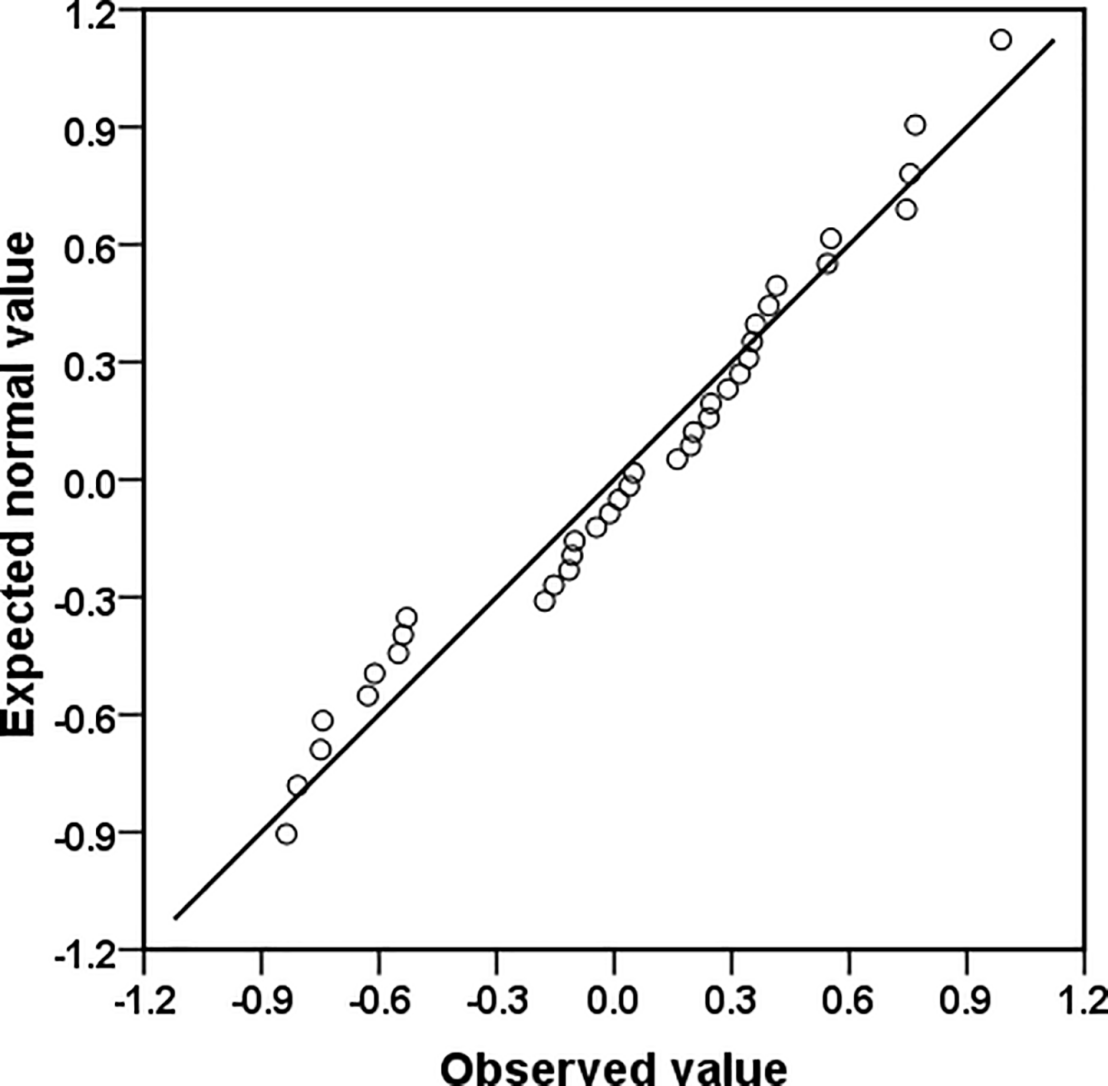
Normal q-q plot of the district-level residuals.

In SAE applications, generally two types of diagnostics measures are suggested and employed, the model diagnostics and the diagnostics for the small area estimates, see [12, 27]. The model diagnostics are applied to verify the assumptions of the underlying model. In equation number (1), the random district (or area) specific effects *u*_*d*_ are assumed to have an independent and identical normal distribution with mean zero and constant variance *ϕ*. Fig 1 shows normal q-q plot of the district level residual, which provides evidence in support of the normality assumption for the district-level residuals.

Besides, this visual method for checking normality, we also perform Shapiro-Wilk (SW) test of normality (i.e., test based on uncertainty measurement in terms of p-value). The p-value from SW test indicates the chance that the sample comes from a normal distribution. Typically, a value of 0.05 is used as cutoff, i.e. if p-value is less than 0.05 we can conclude that the sample deviates from normality. We use *shapiro*.*test()* function in R software to implement this test. The SW test (W = 0.968 and p-value = 0.330) result with large p-value confirming the normality of district-level residuals. These results clearly indicate that the normality assumption is satisfied reasonably well for the data.

Other diagnostics are used to examine reliability (and validity) of the model-based small area estimates. Such diagnostics are suggested in [27]. The model-based small area estimates should be consistent with the unbiased direct survey estimates, be more precise than the direct survey estimates, and provide reasonable results to users. The values for the model-based small area estimates derived from the fitted model should be consistent with the unbiased direct survey estimates, wherever these are available, i.e. they should provide an approximation to the direct survey estimates that is consistent with these values being "close" to the expected values of the direct estimates. The model-based small area estimates should have mean squared errors significantly lower than the variances of corresponding direct survey estimates. For this purpose, we consider three commonly used measures, a bias diagnostic, a percent coefficient of variation (CV) diagnostic, and a 95 percent confidence interval-diagnostic. See for example, [12, 13, 27], for more information.

**Fig 2:**
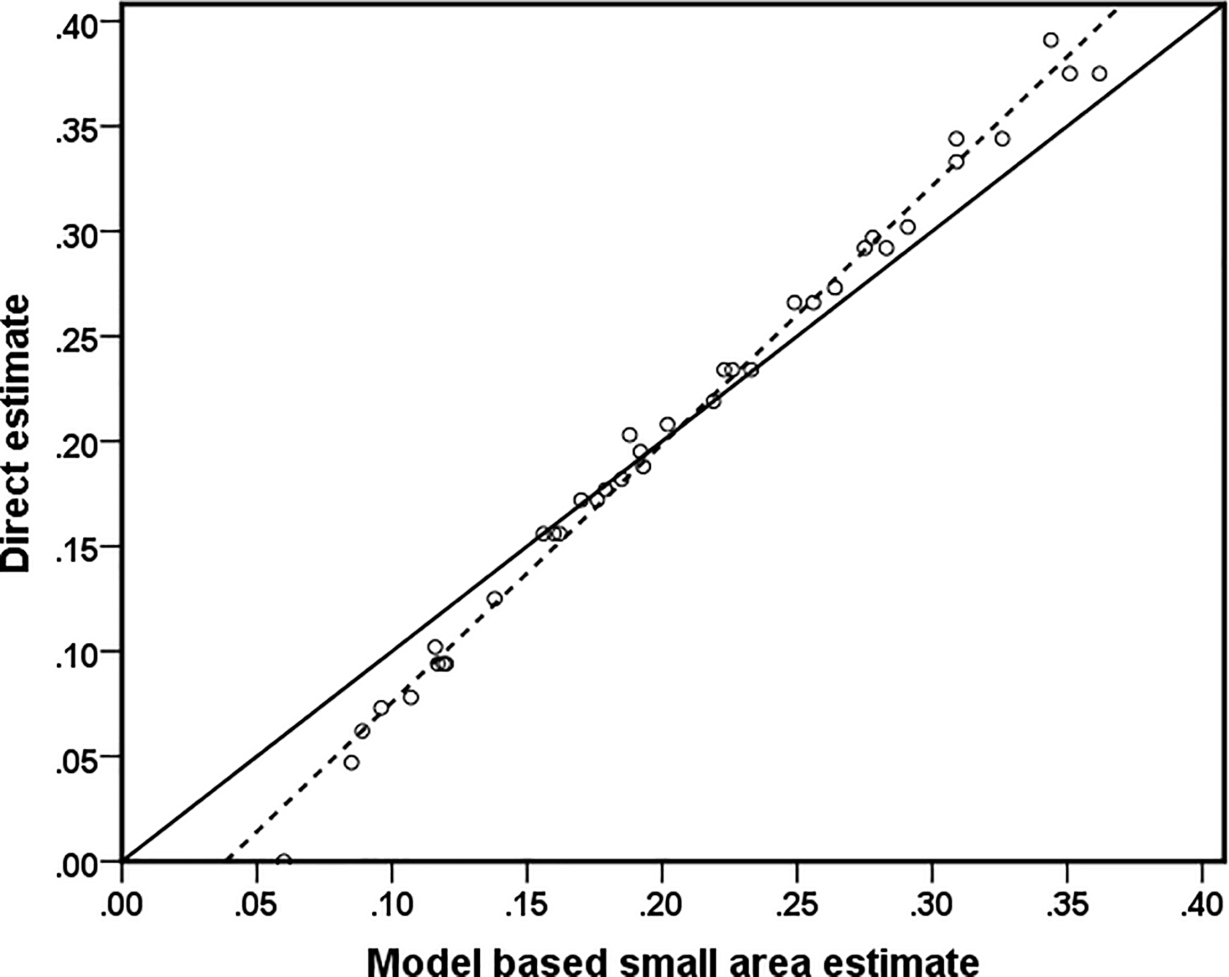
Bias diagnostics plots with *y* = *x* line (solid line) and regression line (dotted line) model based small area estimate.

The bias diagnostic is used to examine if the model-based small area estimates are less extreme when compared to the direct survey estimates, when it is available. If direct survey estimates are unbiased, their regression on the true values should be linear and correspond to the identity line. Further, if model-based small area estimates are close to the true values the regression of the direct survey estimates on these model-based estimates should be similar. We therefore plot direct survey estimates on the y-axis and corresponding model-based small area estimates on x-axis and we look for divergence of the fitted least squares regression line from the *y* = *x* and test for intercept = 0 and slope = 1. In particular, the aim of the diagnostic is a simple test that the straight line found by regressing the direct estimate against the model-based estimate provides an adequate fit of the small area estimates. The bias scatter plot of the district level direct survey estimates against the corresponding model-based small area estimates is given in Fig 2, with fitted least squares regression line (dotted line) and line of equality (solid line) superimposed. The bias diagnostic plot in Fig 2 indicates that the district level model-based estimates generated by EBP are less extreme when compared to the direct survey estimates, demonstrating the typical SAE outcome of shrinking more extreme values towards the average. The estimates of poverty incidence generated by EBP method lies close to the line y = x for most of the districts, which indicates that they are approximately design unbiased. This is expected, since the EBP estimates are realisation of random variables and so the regression of the direct estimates on the EBP estimates is unbiased for a test of common expected values. Such a test is provided by the Goodness of Fit (GoF) diagnostic.

This diagnostic tests whether the direct estimates and the model-based estimates generated by EBP are statistically different. The null hypothesis is that the direct estimates and the model-based estimates are statistically equivalent. The alternative is that the direct estimates and the model-based estimates are statistically different. The GoF diagnostic is computed using the following Wald statistic for EBP estimate:

$$W=\sum_{d}\left\{\frac{{\left(\mathrm{Direct}\;\mathrm{estimat}e_{d}‑\mathrm{EBP}\;\mathrm{estimat}e_{d}\right)}^{2}}{\hat{Var}(\mathrm{Direct}\;\mathrm{estimat}e_{d})+\hat{MSE}(\mathrm{EBP}\;\mathrm{estimat}e_{d})}\right\}.$$

The value from the test statistic is compared against the value from a chi square distribution with *D* degrees of freedom. The value of chi square statistic with *D = 38* degrees of freedom is 24.88 at 5% level of significance. In this analysis, the value of Wald statistic is W = 11.81. A smaller value (less than 24.88 in this case) indicates no statistically significant difference between the direct estimates and the model-based estimates generated by EBP. The diagnostic results clearly show that the model based small area estimates are consistent with the direct survey estimates.

In small area applications, aggregation or benchmarking of small area estimates at higher level is always desirable by the users. National statistical offices involved in generating the small area estimates always expect that the small area estimates are aggregated/ benchmarked to higher level estimate. At higher level of aggregation, the direct estimates are considered to be reliable and therefore the model-based small area estimates are expected to be near to the direct estimates when they are aggregated. We checked the aggregation of model-based small area estimates at state level. We computed state level incidence of poverty by aggregating the direct estimates and the model-based small area estimates (i.e. EBP), as ∑_*d*_(*N*_*d*_×Direct estimate_*d*_)/∑_*d*_*N*_*d*_ and ∑_*d*_(*N*_*d*_×EBP estimate_*d*_)/∑_*d*_*N*_*d*_, respectively. The state level estimate of incidence of poverty computed by aggregation of direct and EBP methods are 0.200 and 0.202 respectively. As one expects, the model-based estimates aggregate well to state level direct estimate.

**Fig 3:**
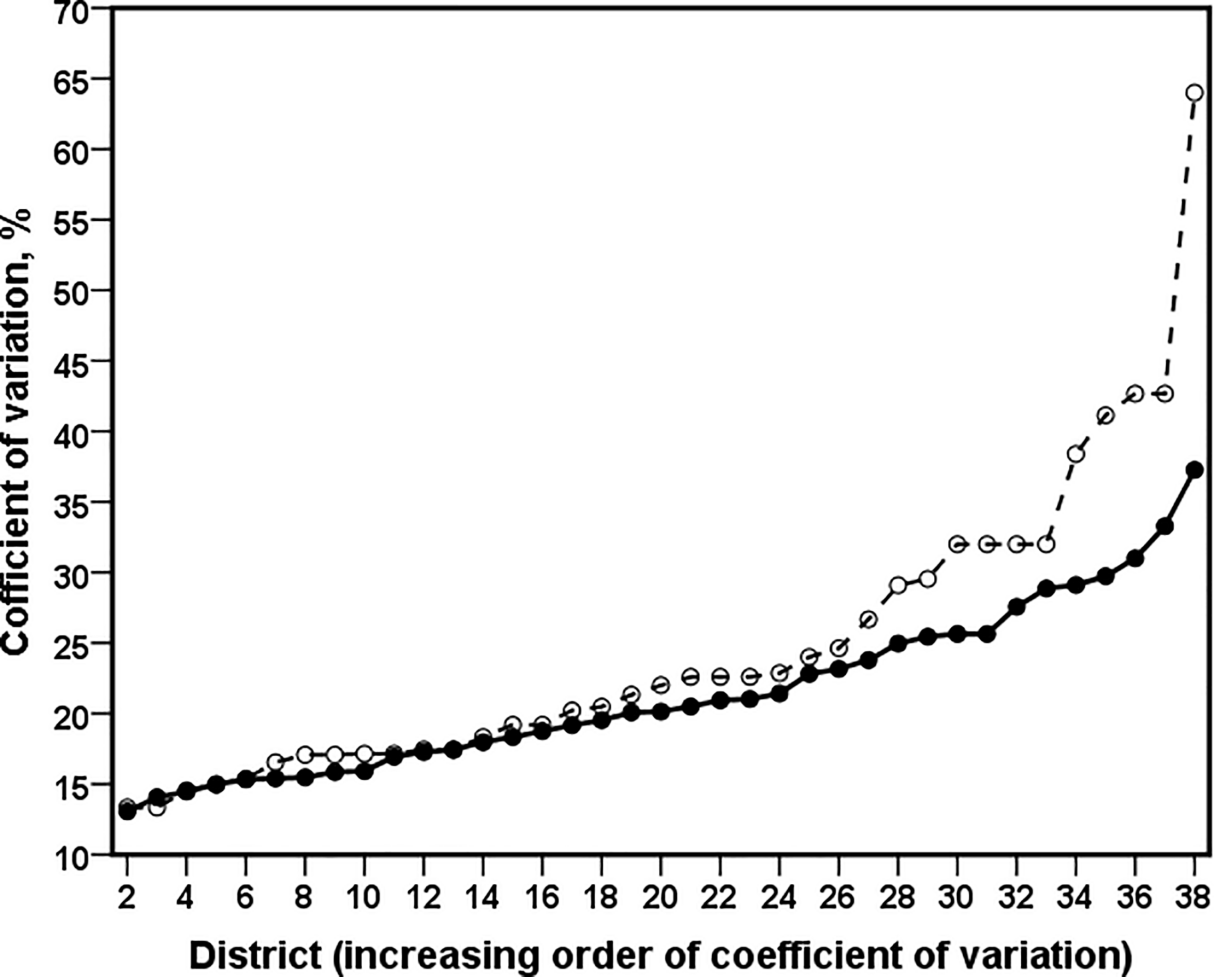
District-wise percentage coefficient of variation for the direct (dash line,°) and model based small area estimate (solid line, •).

We use the percent CV to assess the improved precision of the model-based small area estimates (EBP) and the direct survey estimates. The CVs show the sampling variability as a percentage of the estimate. Estimates with large CVs are considered unreliable (i.e. smaller is better). In general, there are no internationally accepted tables available that allow us to judge what is "too large". Different organization uses different cut off for CV to release their estimate for the public use. For example, some country uses cut off CV value of 20% for acceptable estimates [13]. However, the CV value of 20% is not standard in all the countries. The % CV of the direct and the EBP estimates are given in Table 1. Fig 3 presents the district-wise distribution of % CV of the model-based estimates generated by EBP and the direct estimates. The results in Table 1 and district-wise values in Fig 3 clearly show that the direct estimates of the proportion of poor households within each district are unstable, with CVs varying from 13.33% to 64% with average of 24.69%. The CVs of the EBP estimates are ranging from 12.96% to 37.27% with average of 21.19%. In Fig 3 and Table 1 we further notice that the CVs of the direct estimates are greater than 20% (30%) in 22 (9) out of the 38 districts. However, the model based estimates are greater than 20% (30%) in 20 (3) out of the 38 districts. The estimate of poverty incidence of Madhepura district is zero because the sample count is zero. As a result, the standard error of direct estimate is zero and hence CV cannot be computed for this district. This is one of the drawback of direct estimation. Except in two districts (i.e. Samastipur and Jamui), the model-based estimates are less variable (i.e. smaller CV), and hence relatively more precise than the direct estimates. In these two districts (Samastipur and Jamui) the value of CVs are at par for both direct and model-based estimates. Overall, using SAE improves the precision of the small area estimates.

The districts-wise 95 percent confidence interval (95% CI) of the EBP and the direct estimates are given in Table 1. It is important to note that the 95% CI for the direct estimates are calculated assuming a simple random sample generated the simple proportions. This ignores the effects of differential weighting and clustering within districts that would further inflate the true standard errors of the direct estimates. The 95% CIs for the model-based estimates are more precise and contain both direct and model-based estimates of the poverty incidence.

**Fig 4:**
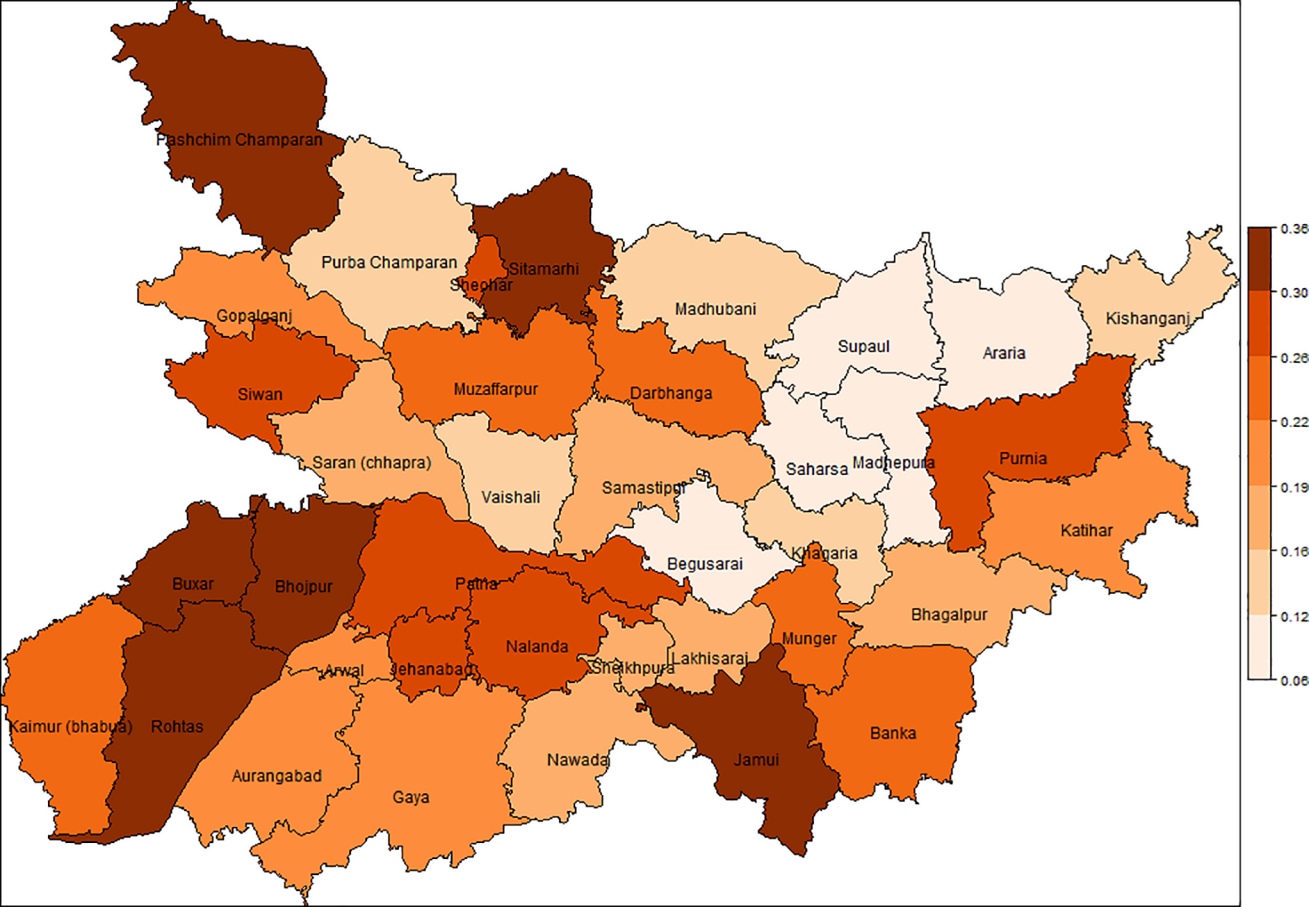
Poverty mapping generated for the state of Bihar in 2011–12.

The spatial mapping of district-wise poverty incidence generated EBP method for the State of Bihar is shown in Fig 4. This map provides the spatial inequality in distribution of poverty incidence, i.e. the degree of inequality with respect to distribution of poor households in different districts. This map is very useful in identifying the districts and regions with low and high level of poverty incidence in the state. The district-wise poverty incidence generated by the EBP method in rural areas of Bihar ranges from 6 to 36% with average of 21%. From Fig 4 and Table 1, it can be seen that Madhepura (6%) has lowest poverty in the state. Supaul, Begusarai, Araria, Saharsa, Madhubani, Vaishali, Kishanganj, Khagaria and Purba Champaran have smallest poverty rate (9–14%) whereas Buxar, Rohtas, Pashchim Champaran, Jamui, Bhojpur and Sitamarhi have highest rate of poverty (31–36%). This map clearly shows that districts bordering with eastern Uttar Pradesh have higher poverty incidence. The district level estimates as well as the spatial map of poverty rates are expected to provide invaluable information to policy-analysts and decision-makers for identifying the regions and districts requiring more attention in the state. This is an example of a "poverty map" showing reliable estimates of poverty incidence across a region of interest.

## Conclusions

Theory of SAE method for estimation of proportions is well developed, however, its application in the field of agricultural or social sciences are not so popular. In developed countries like USA, UK, Australia etc., SAE has been initiated and included as a part of their objectives in the national statistical offices. Although need of small area statistics has been felt in different agencies and organization in India, but, not much initiative has been taken place. In India, the Census is usually limited in its scope in collection of data; it focuses mainly on basic social and demographic information and that too at decennial interval. On the other hand, NSSO conducts regular surveys on a number of socioeconomic indicators, but their utility is restricted to generate national and state level estimates, but not administrative units below state because of small sample sizes for such units. This paper demonstrates that the SAE can be used as cost effective and efficient approach for generating fairly accurate disaggregate level estimates of the poverty incidence from existing survey data and using auxiliary information from different published data sources. The results clearly indicates the advantage of using SAE technique to cope up the small sample size problem in producing reliable estimates. Notably, the model-based SAE method brings gain in efficiency in district level estimates of the poverty.

Disaggregate level estimates of poverty incidence and poverty map are useful information for identifying the districts/regions with higher level of poverty rate. In particular, the poverty map shows how household poverty incidence varies by district across the State of Bihar in India. This type of map is a useful aid for policy planners and administrators charged with taking effective financial and administrative decisions that can impact differentially across the region. We conclude by observing that the estimates and spatial distribution of poverty incidence generated from this research should be useful for meeting the data requirements for policy research and strategic planning by different international organizations and by Departments and Ministries in the Government of India. These information can be used by state government of Bihar in allocation of budget in various government schemes. The methodology including MSE estimation and application to real data presented in this paper can also be used for producing reliable, timely and cost effective estimates using survey data from different sectors.

## Supporting information

S1 File Population census 2011 data—Bihar. (XLSX)

S2 File NSSO data district aggregate—Bihar. (XLSX)
